# Supplementary material for: Poly-γ-glutamylation of biomolecules
Source: Nat Commun. 2024 Feb 12;15:1310. doi: 10.1038/s41467-024-45632-1 (PMC10861534; doi:10.1038/s41467-024-45632-1)
Supplement: Supplementary file 1 — Supplementary information [file 41467_2024_45632_MOESM1_ESM.pdf]

Supplementary Information

Poly-γ-glutamylation of biomolecules

Ghader Bashiri[1,2*], Esther M. M. Bulloch[1,2], William R. Bramley[1], Madison Davidson[3], Stephanie M. Stuteley[1,2], Paul G. Young[1,2], Paul W. R. Harris[1,2], Muhammad S. H. Naqvi[1], Martin J. Middleditch[1], Michael Schmitz[4], Wei-Chen Chang[3], Edward N. Baker[1,2], Christopher J. Squire[1,2*]

[1]School of Biological Sciences, The University of Auckland, Private Bag 92019, Auckland 1142, New Zealand

[2]Maurice Wilkins Center for Molecular Biodiscovery, The University of Auckland, Private Bag 92019, Auckland 1142, New Zealand

[3]Department of Chemistry, North Carolina State University, NC 27695-8204, USA

[4]School of Chemical Sciences, The University of Auckland, Private Bag 92019, Auckland 1142, New Zealand

*Correspondence to: g.bashiri@auckland.ac.nz and c.squire@auckland.ac.nz

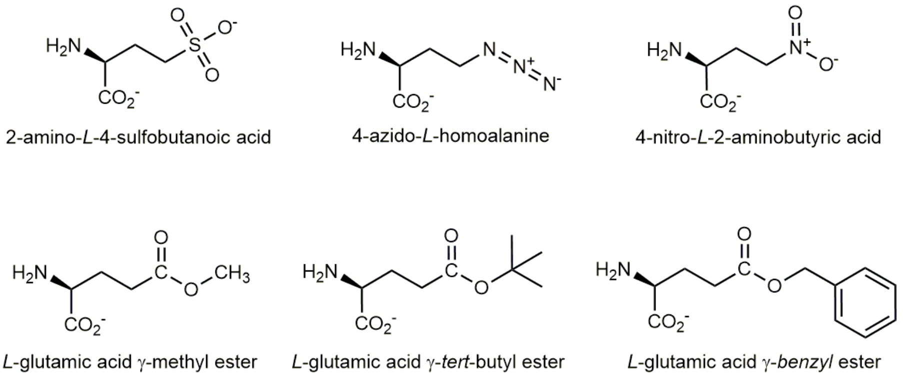

Supplementary Fig. 1. L-glutamate analogues used in this study. Each analogue was assayed in biochemical reactions for poly-γ-glutamylation of folate and F_{420} substrates paired with human or *M. tuberculosis* FPGS, *M. tuberculosis* or *Archaeoglobus fulgidus* γ-glutamyl ligases, as appropriate. 5-Nitro-L-2-aminobutyric acid was the only molecule shown by LC-MS to act as both substrate and a terminator of glutamylation.

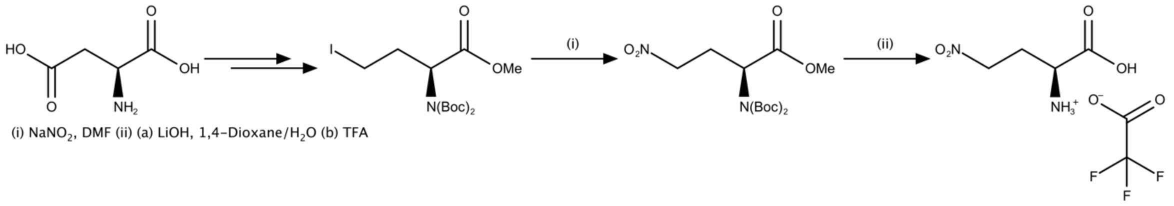

Supplementary Fig. 2. Preparation of (2*S*)-2-amino-4-nitrobutanoic acid.

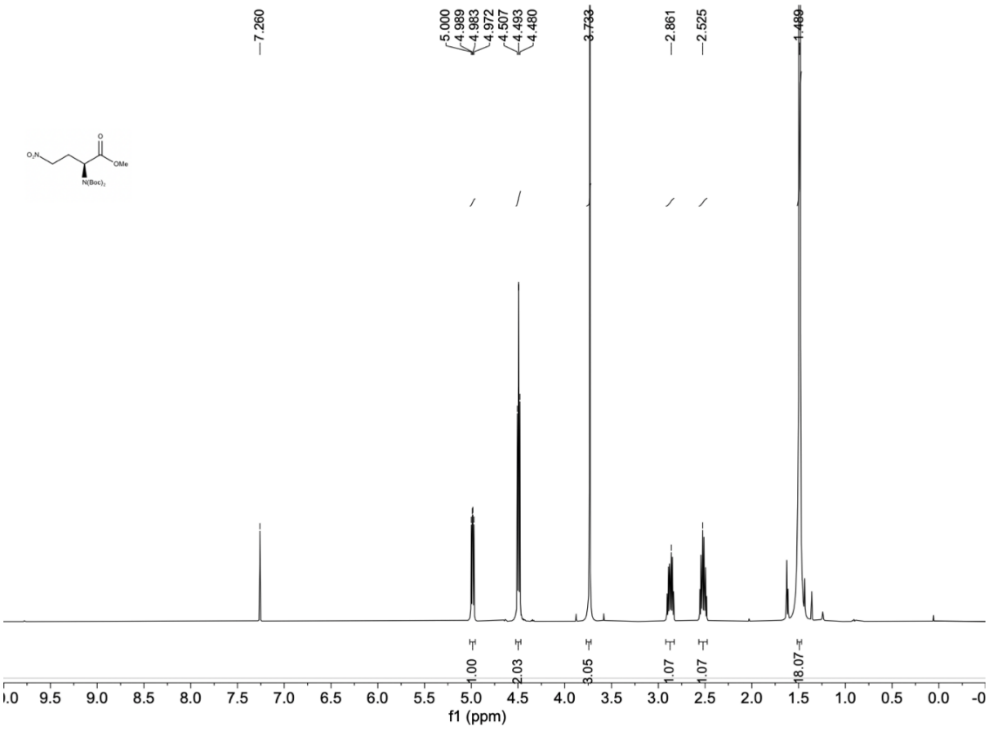

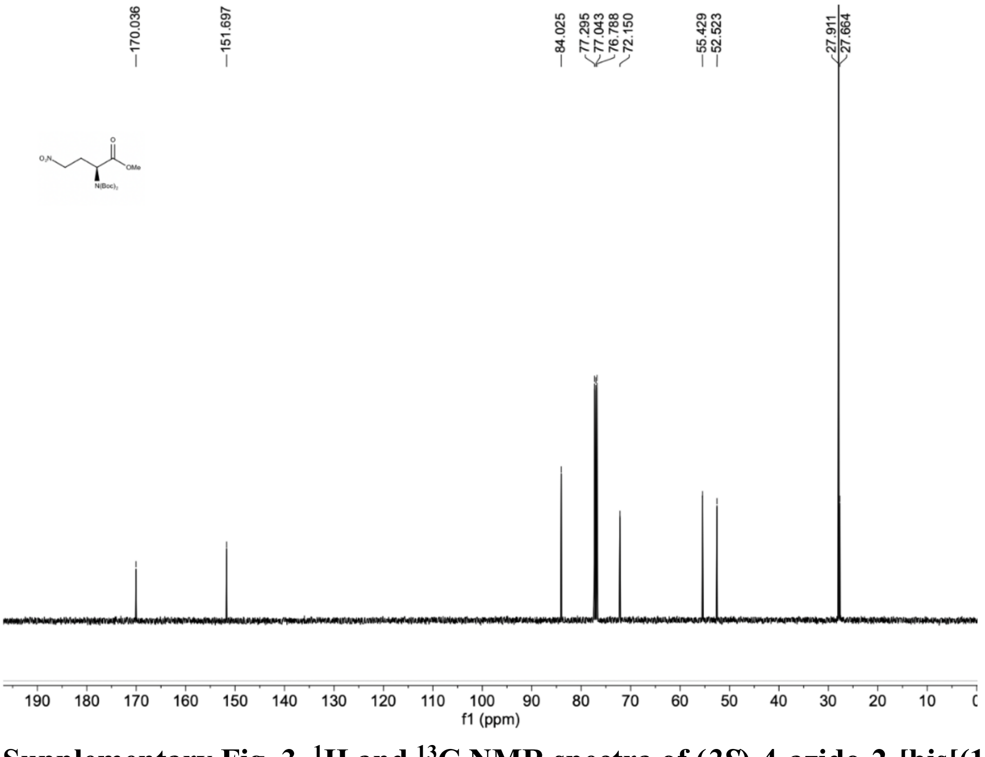

Supplementary Fig. 3. ^{1}H and ^{13}C NMR spectra of (*2S*)-4-azido-2-[bis[(1,1-dimethylethoxy)carbonyl]amino]-butanoic acid methyl ester.

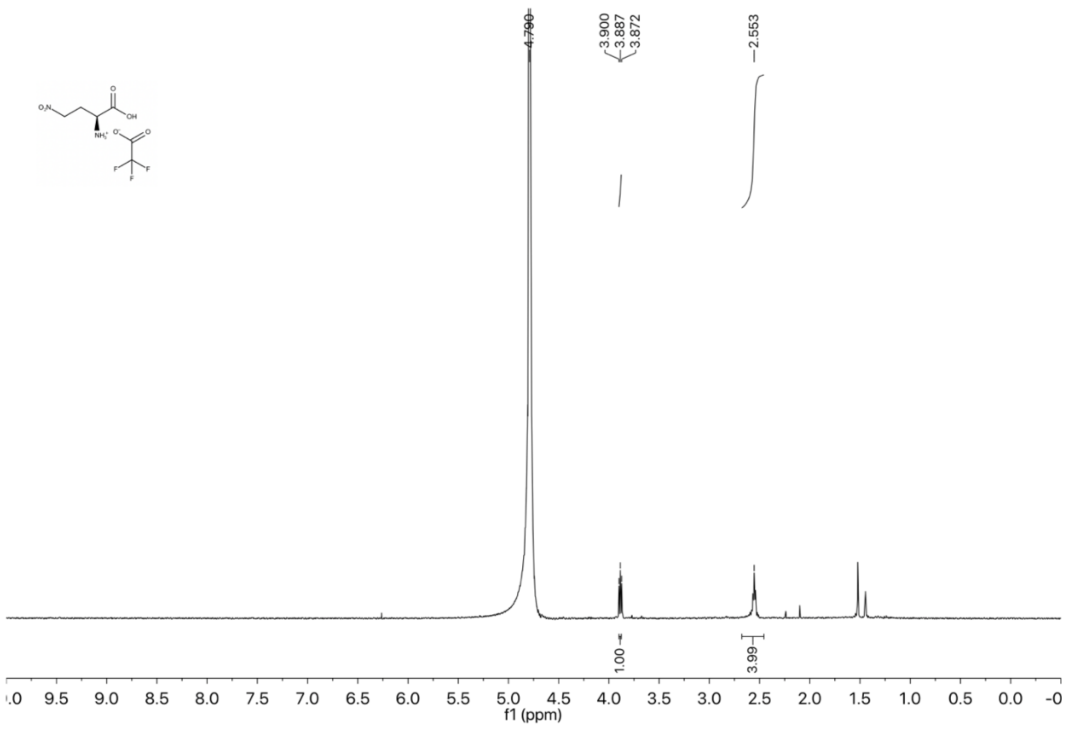

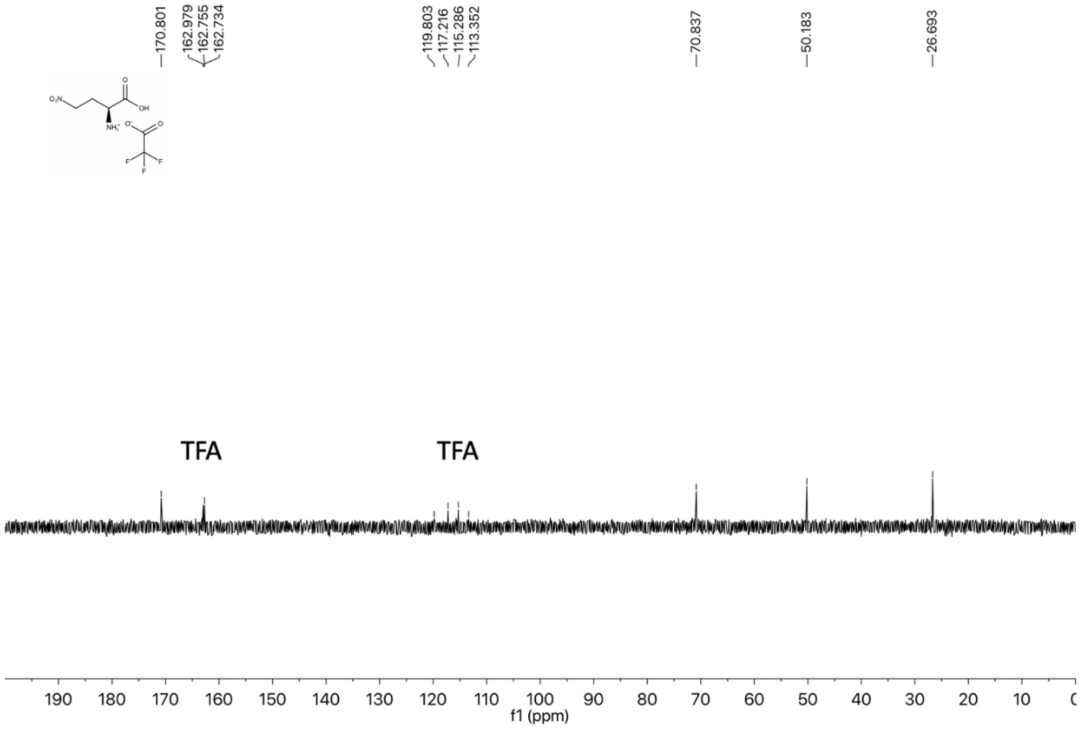

Supplementary Fig. 4. ^{1}H and ^{13}C NMR spectra of (*2S*)-2-amino-4-azidobutanoic acid.

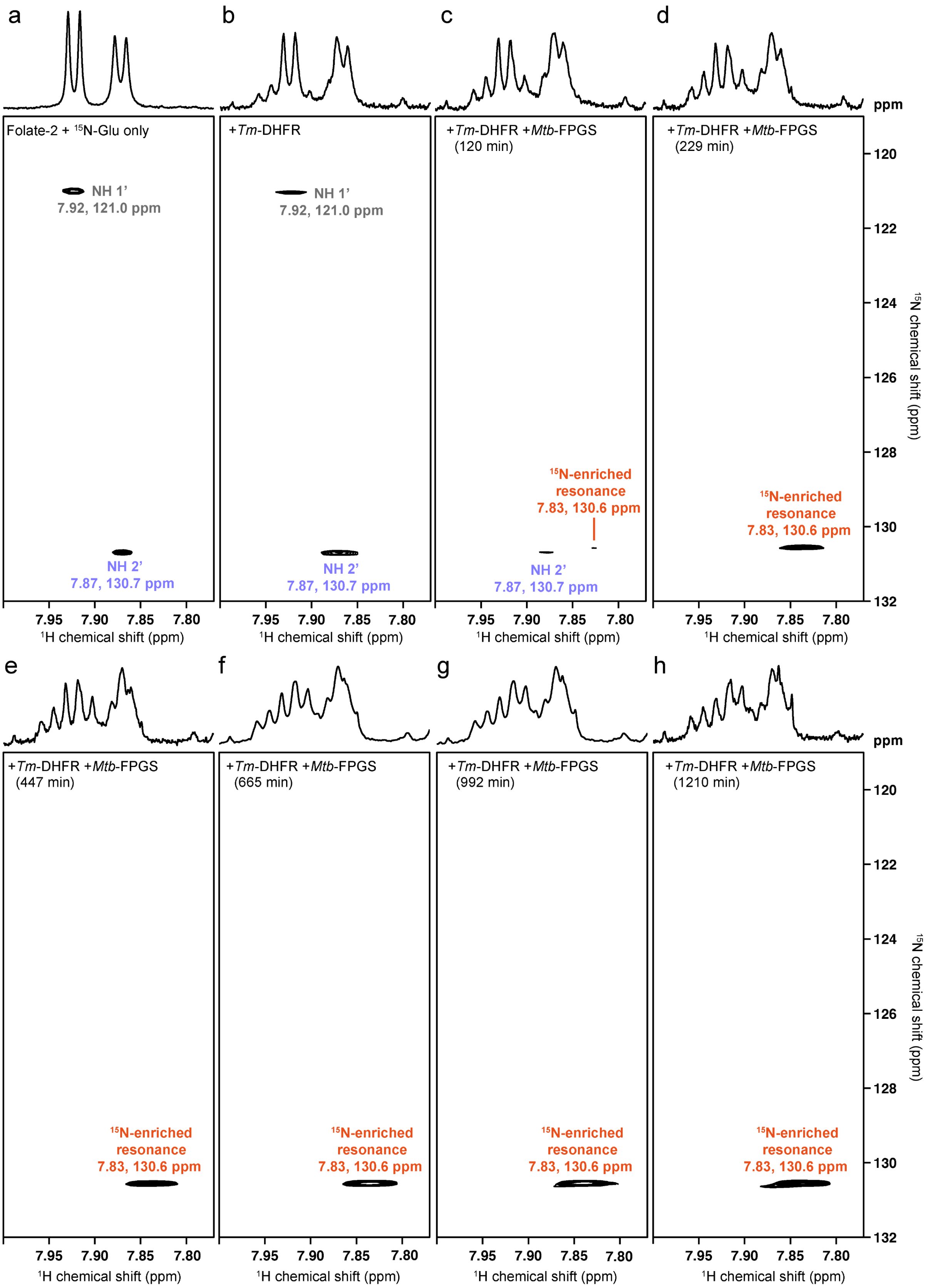

Supplementary Fig. 5. *Mtb*-FPGS-dependent glutamylation of folate-2 with ^{15}N-glutamate monitored by NMR spectroscopy. 1D ^{1}H and 2D ^{1}H-^{15}N HSQC spectra for reaction samples as follow. (a) *Mtb*-FPGS/*Tm*-DHFR reaction sample prior to addition of

enzyme, (b) after a 120 min pre-incubation with *Tm*-DHFR, and (c to h) at time points (as indicated) following the further addition of *Mtb*-FPGS enzyme.

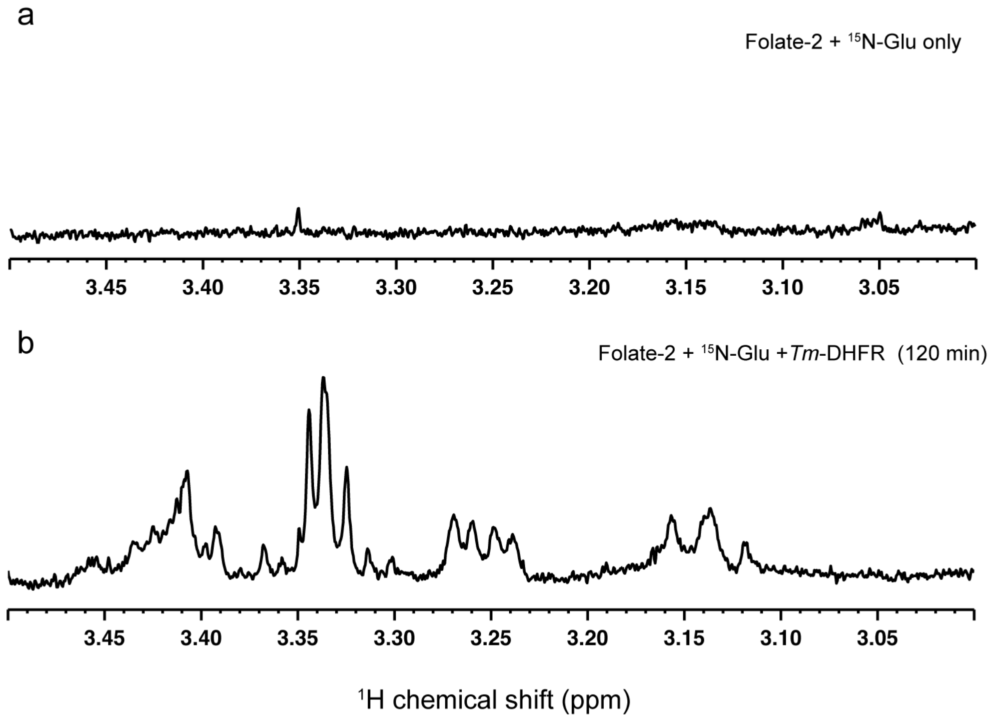

Supplementary Fig. 6. *Mtb*-FPGS-dependent production of tetrahydrofolate-3 from folate-2 monitored by 1D ^{1}H NMR spectroscopy. Selected region of 1D ^{1}H NMR spectra for (a) reaction sample prior to addition of enzyme, and (b) after a 120 min pre-incubation with *Tm*-DHFR. The ^{1}H multiplet peaks centered at 3.14, 3.25, 3.34 and 3.41 ppm arise from the tetrahydropterin of tetrahydrofolate-2 [18].

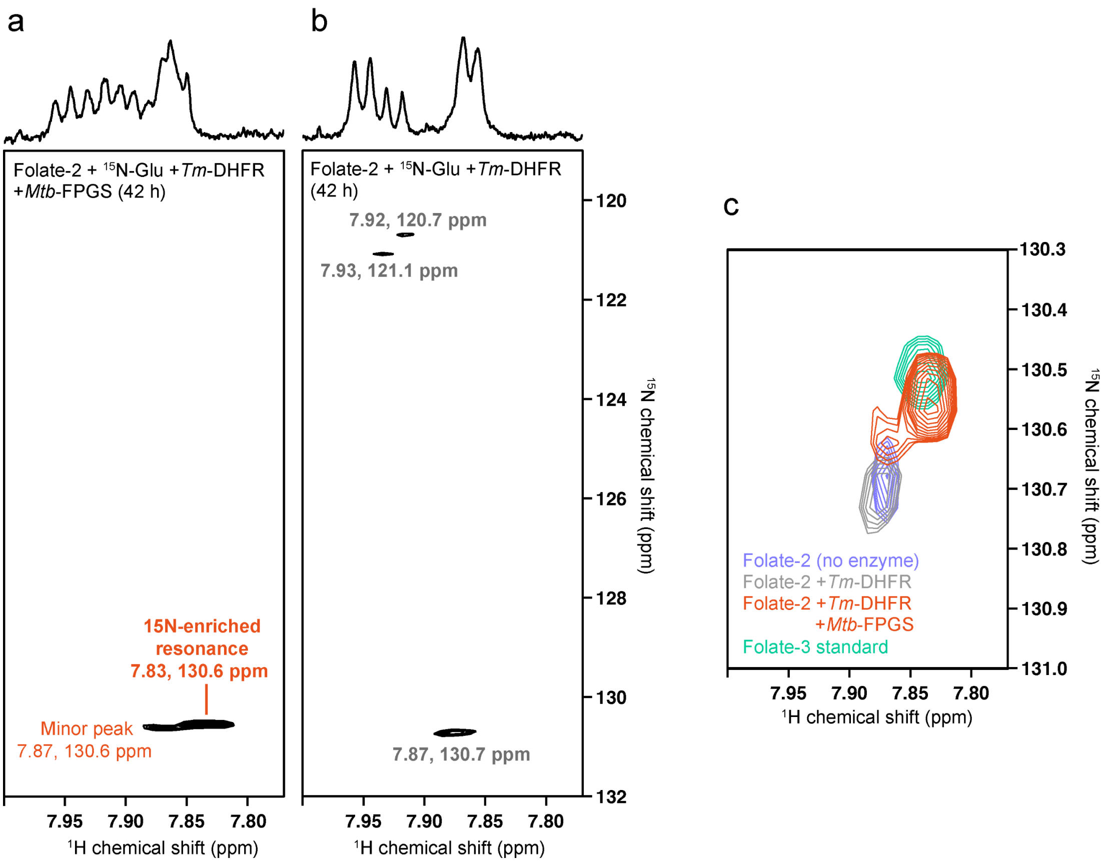

Supplementary Fig. 7. Comparison of final ^{1}H-^{15}N HSQC spectra for *Mtb*-FPGS/*Tm*-DHFR coupled reaction and a *Tm*-DHFR only control reaction. Higher sensitivity ^{1}H-^{15}N HSQC experiments were run from 22 to 42 h post-initiation with enzyme on the (a) *Mtb*-FPGS/*Tm*-DHFR coupled reaction sample and (b) *Tm*-DHFR only control. (c) Overlay and expansion of the ^{1}H-^{15}N HSQC spectra of a reaction sample prior to addition of enzyme (blue), final *Tm*-DHF-only control reaction (grey), final *Mtb*-FPGS/*Tm*-DHFR reaction (orange) and a folate-3 standard (green).

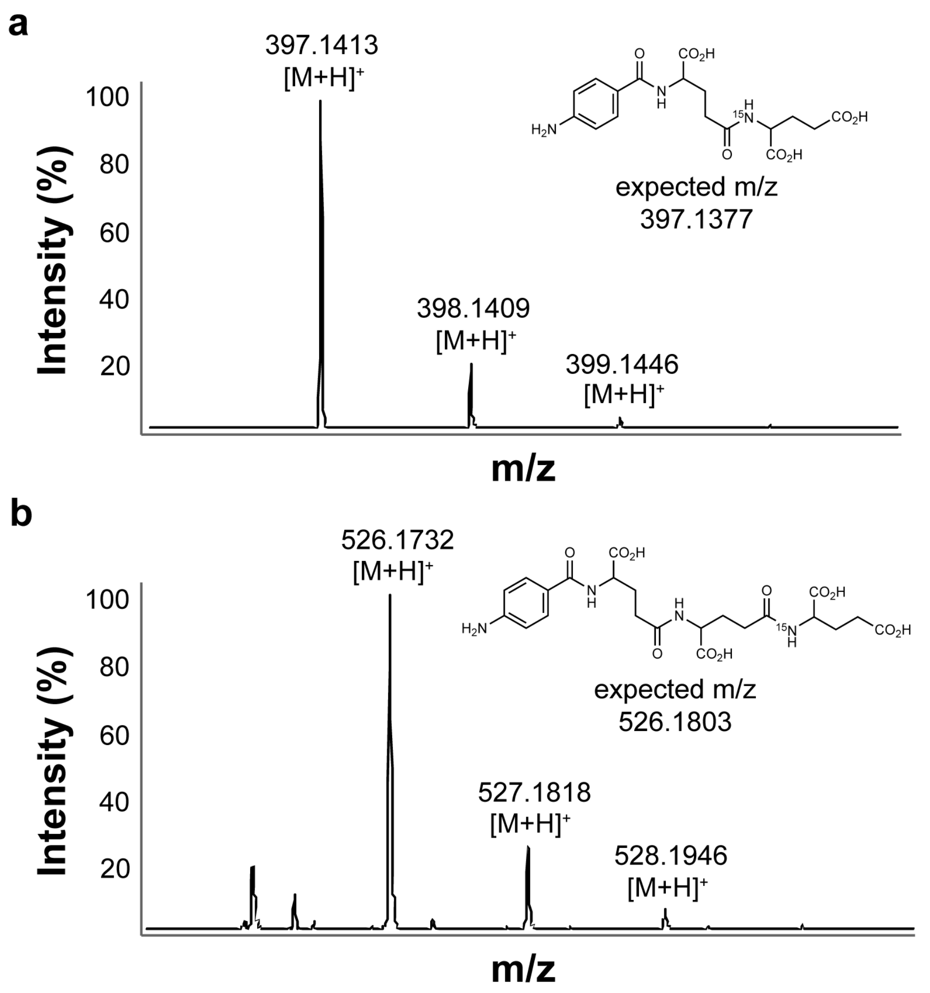

Supplementary Fig. 8. LC-MS analysis of *Mtb*-FPGS/*Tm*-DHFR coupled reaction. (a) Mass spectrum of *N*-(4-aminobenzoyl)-(L-Glu)$_2$, degradative product of tetrahydrofolate-2. (b) Mass spectrum of *N*-(4-aminobenzoyl)-(L-Glu)$_3$, degradative product of tetrahydrofolate-3. Both molecules have resulted from spontaneous oxidation of their respective tetrahydrofolate parent molecules. Carboxylic acids are shown in their protonated form as appropriate for mass analysis in positive ion mode and include an ^{15}N isotope in their terminal glutamic acid moieties.

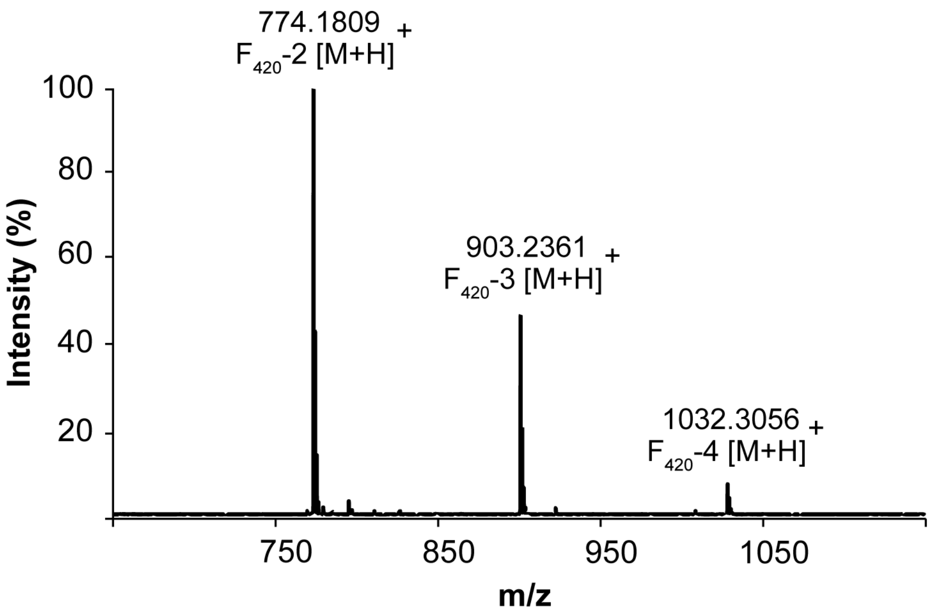

Supplementary Fig. 9. LC-MS analysis of CofE modification of F_{420}. The mass spectrum analysis conducted in the positive ion mode shows the predominant species produced is F_{420}-2 (expected mass 773.1793), but with larger F_{420}-3 (expected mass 902.2219) and F_{420}-4 (expected mass 1031.2645) species clearly present.

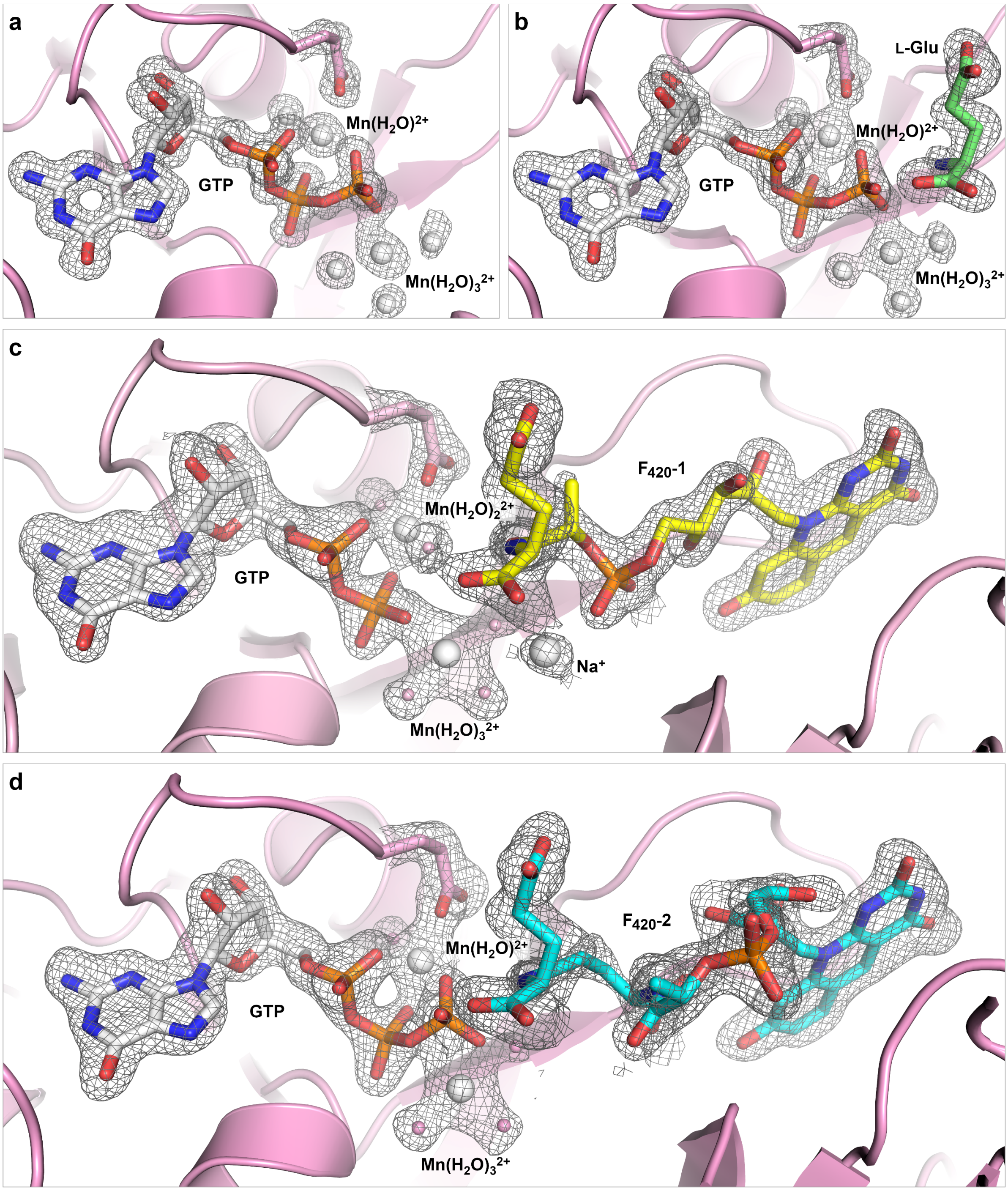

Supplementary Fig. 10. Unambiguous localization of substrates, cofactors, and metal ions in the CofE crystal structures. (a) Electron density and placement of GTP and water coordinated manganese metals in the GTP-only CofE structure. (b) L-glutamate binds adjacent to the GTP terminal phosphate in the primary glutamate site in the GTP/L-glutamate CofE structure. (c) F_{420}-1 binding shows metal coordination and the presence of a monovalent cation (Na^+). The substrate terminal glutamyl occupies the identical site to free L-glutamate. (d) F_{420}-2 binds near identically to F_{420}-1 at each terminus (isoalloxazine and terminal glutamyl) but with the base glutamyl occupying the F_{420}-1 phospholactyl or secondary glutamate site. The F_{420}-2 ribityl and phospholactyl bulge out toward solvent to accommodate

the growing tail. In each figure part, $2F_o$-F_c electron density maps are shown as the 1σ level and were calculated prior to the refinement of ligand molecules.

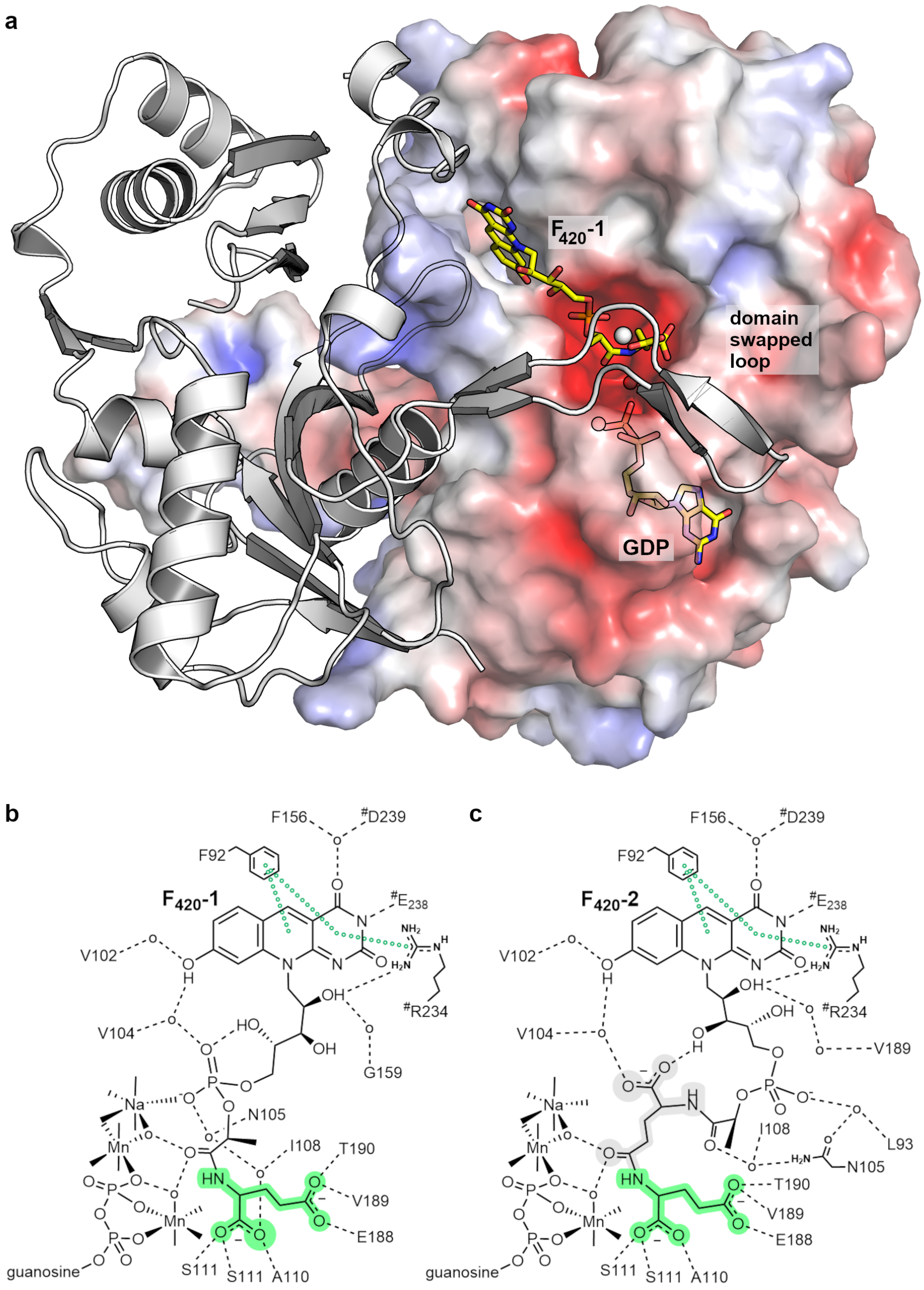

Supplementary Fig. 11. Structure of CofE highlighting ligand binding. (a) The structure of the CofE dimer – the crystallographic asymmetric unit contains a single molecule (surface model) with the dimer (cartoon model) produced using a symmetry transformation. F_{420}-1, GDP, and metal ions occupy a long cavity located at the dimer interface. A domain-swapped loop completes the active site, forming specific contacts to either free L-glutamate or terminal glutamyls of F_{420}-1 or F_{420}-2. (b) Intermolecular contacts between F_{420}-1 and CofE. Polar interactions are shown as dashed black lines and selected water molecules by small "o" symbols. Ring-stacking and cation-π stacking interactions are highlighted by green dots. A complex GDP and metal coordination site makes direct and water-mediated contacts to the substrate molecule. (c) The equivalent binding by F_{420}-2 includes specific contacts that stabilize the bulged tail configuration including an internal H-bond between the base

glutamyl α-carboxylate and the second ribityl hydroxyl group. In both F_{420}-1 and F_{420}-2, water-mediated interactions between the glutamyl bearing tail and the isoalloxazine ring further stabilize the substrate conformation.

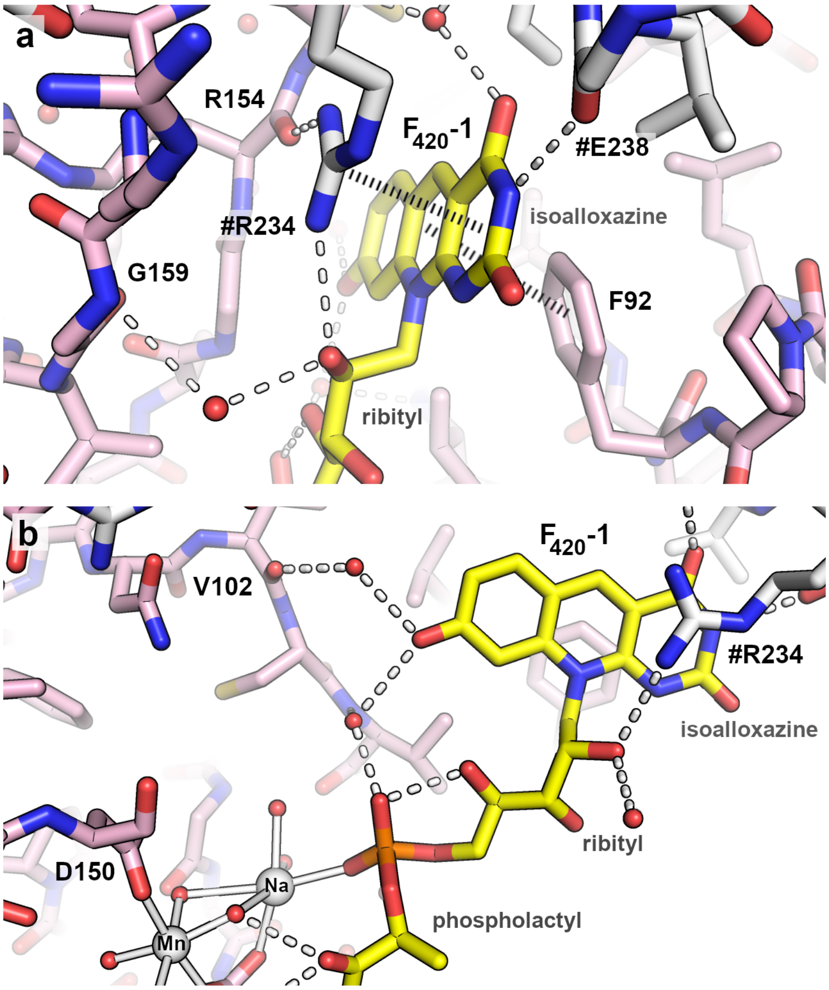

Supplementary Fig. 12. F_{420}-1 binding interactions in CofE. (a) Isoalloxazine binding showing the burial of the chromophore ring, polar interactions as dashed white lines, and ring-stacking and cation-π stacking as black hashed lines. The dimer pair #R234 side chain as shown links monomers via the backbone of R154 and also provides polar and stacking interactions to the F_{420}-1 molecule. (b) Isoalloxazine, ribityl, and phospholactyl segments form numerous binding interactions including direct and water mediate interactions with manganese and sodium cations – the second manganese cation coordinating GDP is not shown.

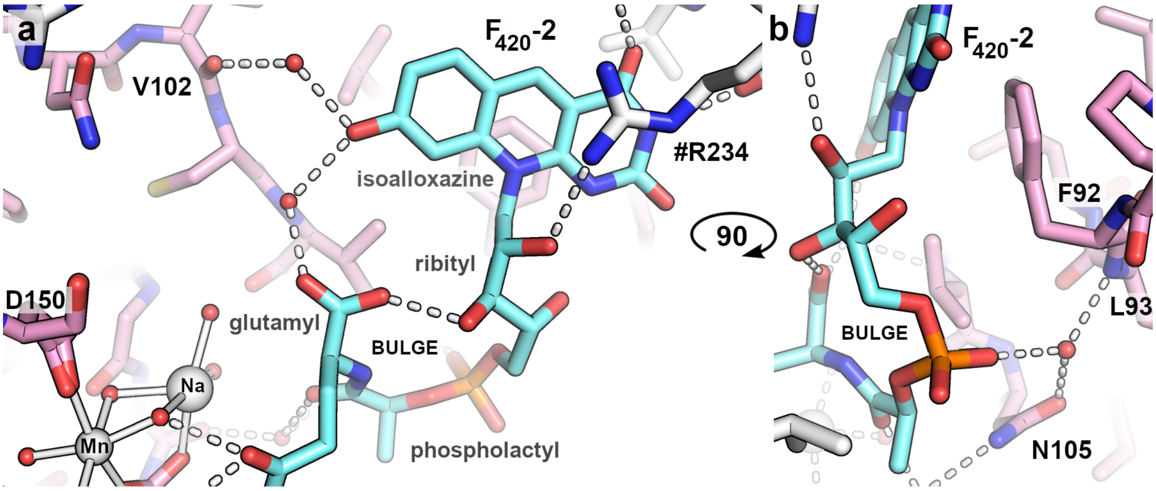

Supplementary Fig. 13. F_{420}-2 binding interactions in CofE. (a) Isoalloxazine binding is near identical to that of F_{420}-1 but the larger glutamyl tail affords new interaction patterns between CofE and ribityl, phospholactyl, and base glutamyl segments. An H-bond network links isoalloxazine to glutamyl to ribityl and stabilized the bulged tail conformation. (b) The bulge is further stabilized by phosphate-water-mediated bonding to the backbone of L93 and side chain of N105. Polar interactions as shown as white-coloured dashed lines, water molecules as small red spheres, metal ions as large white spheres, and direct metal coordination by thin white-coloured bonds.

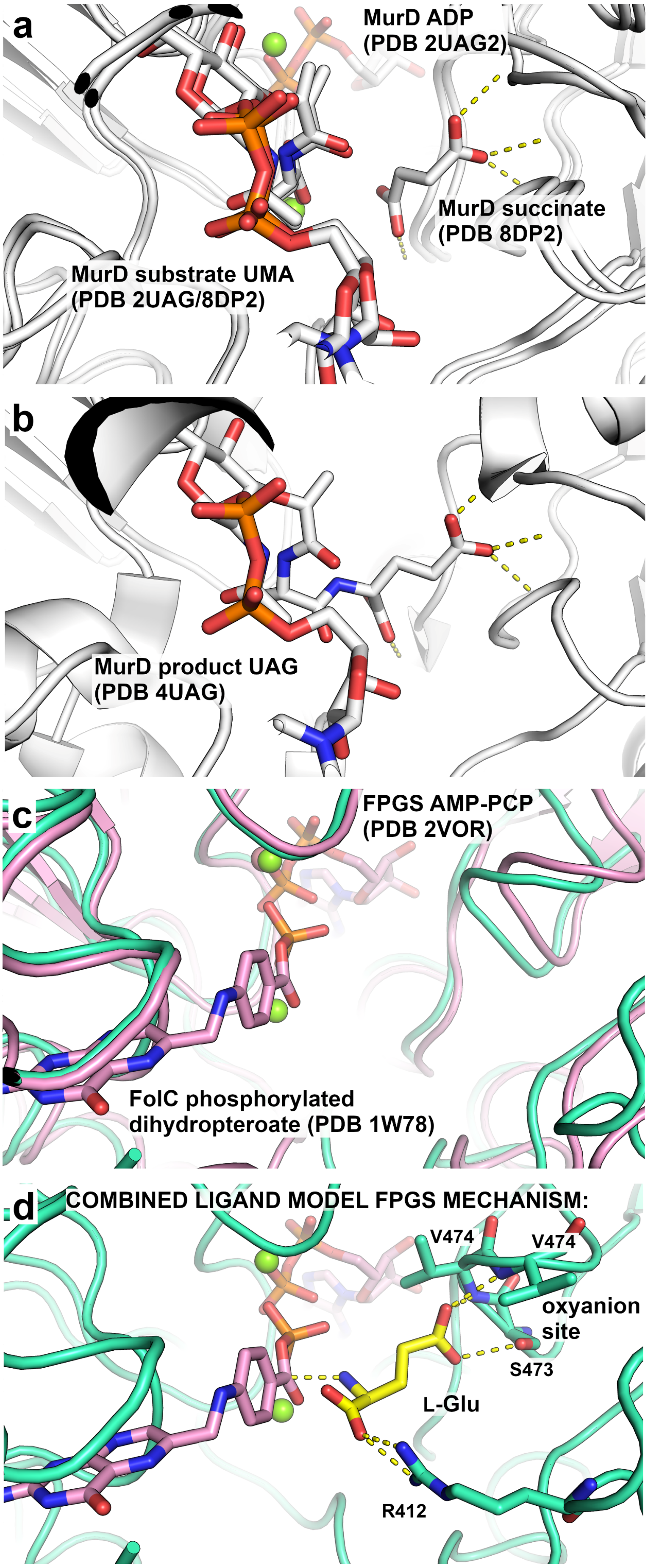
a
MurD ADP
(PDB 2UAG2)
MurD succinate
(PDB 8DP2)
MurD substrate UMA
(PDB 2UAG/8DP2)
b
MurD product UAG
(PDB 4UAG)
c
FPGS AMP-PCP
(PDB 2VOR)
FolC phosphorylated
dihydropteroate (PDB 1W78)
d
COMBINED LIGAND MODEL FPGS MECHANISM:
V474
V474
oxyanion
site
S473
L-Glu
R412

Supplementary Fig. 14. Structural comparison of CofE/F_{420}-2, MurD, FolC, and FPGS systems supporting a conserved mechanism of glutamate ligation. Structures overlaid in this comparison are *E. coli* MurD, UDP-*N*-acetylmuramoylalanine-D-glutamate ligase, (PDB 2UAG; white) [27], *E. coli* FolC, bifunctional dihydrofolate synthase/folylpolyglutamate synthase, (PDB 1W78; pink) [31], and *M. tuberculosis* FPGS, (PDB 2VOR; green) [15]. All structures share the same fold and active site metal and ADP/ATP binding elements. (a) Combined MurD structures 2UAG and 8DP2. The structures display the same substrate and metal binding. A succinate ligand in 8DP2 is a model for D-glutamate which would be appropriately located adjacent to the substrate carbonyl that is modified in the enzymatic mechanism. Polar interactions between succinate and MurD are shown as dashed yellow lines. (b) MurD structure displaying product binding following a single γ-glutamylation event (PDB 4UAG) [27]. Polar interactions between the terminal glutamyl of the product and MurD are shown as dashed yellow lines. (c) Overlaid structure of FolC and FPGS – the structures align with an rmsd of 1.65 Å over 379 Cα atoms. The structures share one divalent metal binding location. The FolC structure contains a phosphorylated folate analogue (phosphorylated dihydropteroate, DHPP) that has been "activated" for γ-glutamylation – the FPGS active site can accommodate this molecule similarly and make attractive interactions. (d) The FPGS active site is shown with ATP binding (background) and modelled phosphorylated folate analogue (DHPP; foreground) derived from the FolC structure [31]. An L-glutamate has been modelled in an adjacent pocket by comparison with succinate or product binding in MurD (PDB 8DP2 or 4UAG, respectively). The hypothetical L-glutamate binding mode positions the amino group adjacent to the folate carbonyl ready for nucleophilic attack and makes a charge-charge interaction between the α-carboxylate and an active site arginine. The γ-carboxylate binds into a putative oxyanion-like hole, formed from a serine and two adjacent backbone NH functional groups and showing a similar geometry to that in the primary glutamate site of our CofE structures. Polar interactions between L-glutamate and FPGS are shown as dashed yellow lines.

Supplementary Table 1. Chemical shift assignments for the polyglutamate chains of folate-2, folate 3 and the ^{15}N-enriched glutamyl unit of the *Tm*-DHFR/*Mtb*-FPGS reaction product at pH 7.0.

Atom No.	Folate-2			Folate-3			^{15}N-enriched glutamyl unit in reaction product	
	^{1}H, ppm	^{13}C, ppm	^{15}N, ppm	^{1}H, ppm	^{13}C, ppm	^{15}N, ppm	^{1}H, ppm	^{15}N, ppm
**1'**	7.92 (d, J = 7.7 Hz)		121.0	7.91 (d, J = 7.7 Hz)		121.0		
**1'α**	4.33 (m)	58.1		4.34 (m)	58.1			
**1'α - CO**		181.4			181.4			
**1'β₁**	2.21 (m),	30.6		2.21 (m),	30.5			
**1'β₂**	2.04 (m)	30.6		2.04 (m)	30.5			
**1'γ**	2.39 (m)	35.3		2.41 (m)	35.3			
**1'δ**		177.9			177.9			
**2'**	7.87 (d, J = 7.5 Hz)		130.7	7.88 (d, J = 7.5 Hz)		130.1		
**2'α**	4.04 (m)	58.1		4.04 (m)	57.8			
**2'α - CO**		181.7			181.0			
**2'β₁**	1.99 (m),	31.2		2.01 (m),	30.7			
**2'β₂**	1.84 (m)	31.2		1.87 (m)	30.7			
**2'γ**	2.16	36.8		2.26 (m)	35.2		2.26†	
**2'δ**		184.9			177.7			
**3'**				7.83 (d, J = 7.7 Hz)		130.5	7.83 (dd, J =91, 7.6Hz)*	130.6
**3'α**				4.09 (m)	58.1		4.09‡	
**3'α- CO**					181.4			
**3'β₁**				2.00 (m),	31.4		2.00‡	
**3'β₂**				1.85 (m)	31.4		1.86‡	
**3'γ**				2.19 (m)	36.8		2.19‡	
**3'δ**					184.7			

* In ^{15}N-decoupled spectra peak collapses to a doublet with J = 7.6 Hz.
† Obtained from ^{1}H-^{1}H NOESY spectra.
‡ Obtained from ^{1}H-^{1}H TOCSY spectra.
Chemical shifts are relative to DSS (δ 0 ppm).

Supplementary Table 2. Data collection and refinement statistics for *Af*-CofE crystal structures containing F_{420}-1/GDP, F_{420}-2/GTP, Glu/GTP, or GTP. All data were collected on the MX2 beamline at the Australian Synchrotron.

	F_{420}-1/GDP structure	F_{420}-2/GTP structure	Glu/GTP structure	GTP structure
**Data collection:**	Australian Synchrotron	Australian Synchrotron	Australian Synchrotron	Australian Synchrotron
**X-ray source**	MX2	MX2	MX2	MX2
**Wavelength**	0.95372	0.95372	0.95370	0.95370
**Resolution[a] (Å)**	48.90 – 1.70 (1.73 – 1.70)	48.30 – 1.83 (1.88 – 1.83)	43.0 – 1.61 (1.64 – 1.61)	46.6 – 1.30 (1.32 – 1.30)
**Space group**	$P\,4_1\,2\,2$	$P\,4_1\,2\,2$	$P\,4_1\,2\,2$	$P\,4_1\,2\,2$
**Cell dimensions**				
**a, b, c (Å)**	69.08, 69.08, 92.35	68.24, 68.24, 91.81	68.71, 68.71, 92.45	68.55, 68.55, 93.28
**α, β, γ (°)**	90.00, 90.00, 90.00	90.00, 90.00, 90.00	90.00, 90.00, 90.00	90.00, 90.00, 90.00
**Unique reflections[a]**	25,326 (1,320)	19796 (990)	29,419 (1,435)	55,332 (2,692)
**Redundancy[a]**	26.7 (27.7)	26.0 (23.6)	7.1 (7.2)	28.0 (27.2)
**Completeness[a] (%)**	100.0 (100.0)	100.0 (100.0)	100.0 (100.0)	100.0 (100.0)
**R_{pim}[ab]**	0.019 (0.653)	0.039 (0.333)	0.035 (0.645)	0.031 (0.693)
**I/σI[a]**	18.2 (1.5)	13.7/1.9	11.7 (1.3)	12.3 (1.2)
**CC1/2[ac]**	0.998 (0.538)	0.998 (0.599)	0.998 (0.544)	0.999 (0.517)
**Refinement:**				
**Resolution (Å)**	48.90 - 1.70	48.30 - 1.83	43.04 - 1.61	43.04 - 1.30
**R_{work}/ R_{free}**	0.203 / 0.231	0.197 / 0.219	0.191 / 0.212	0.157 / 0.184
**No. atoms**				
**Protein**	1862	1952	1894	1919
**Ligands**	28 (GDP), 44 (F4I)	60 (GDP/GTP), 53 (F42)	32 (GTP), 10 (L-Glu)	32 (GTP)
**Water, ions**	136 (HOH), 3 (MN, NA)	126 (HOH), 4 (MN, NA), 5 (SO4)	86 (HOH), 2 (MN)	204 (HOH), 3 (MN, NA), 14 (CO3, SO4)
**Average B-factors (Å^2)**				
**Protein**	39.1	30.4	27.2	18.4
**Ligands**	33.4 (GDP), 35.9 (F4I)	23.8 (GDP/GTP), 34.1 (F42)	19.0 (GTP), 27.5 (NLG)	12.7 (GTP)
**Water, ions**	41.9 (HOH), 33.3 (MN, NA)	35.6 (HOH), 29.0 (MN, NA), 45.3 (SO4)	30.9 (HOH), 20.2 (MN)	31.5 (HOH), 23.0 (MN, NA), 50.2 (CO3, SO4)
**R.m.s deviations**				
**Bond lengths (Å)**	0.006	0.004	0.007	0.006
**Bond angles (°)**	1.386	1.28	1.41	1.38
**Ramachandran favored (%)**	99.0	98.0	98.4	98.0
**Ramachandran outliers (%)**	0	0	0	0
**Molprobity score**	100th percentile	100th percentile	100th percentile	99th percentile
**PDB code**	7ULE	7ULD	7ULF	8G8P

[a]Numbers in parentheses are for the outermost shell. [b]R_{pim} = Precision-indicating *R* factor [51]. [c]CC1/2 = Correlation coefficient [52].
