# Supplementary material for: Poly-γ-glutamylation of biomolecules
Source: Nat Commun. 2024 Feb 12;15:1310. doi: 10.1038/s41467-024-45632-1 (PMC10861534; doi:10.1038/s41467-024-45632-1)
Supplement: Supplementary file 3 — Reporting Summary [file 41467_2024_45632_MOESM3_ESM.pdf]

nature portfolio

Corresponding author(s): Ghader Bashiri, Christopher Squire

Last updated by author(s): Jan 4, 2024

Reporting Summary

Nature Portfolio wishes to improve the reproducibility of the work that we publish. This form provides structure for consistency and transparency in reporting. For further information on Nature Portfolio policies, see our Editorial Policies and the Editorial Policy Checklist.

Statistics

For all statistical analyses, confirm that the following items are present in the figure legend, table legend, main text, or Methods section.

n/a	Confirmed	
☐	☒	The exact sample size (*n*) for each experimental group/condition, given as a discrete number and unit of measurement
☒	☐	A statement on whether measurements were taken from distinct samples or whether the same sample was measured repeatedly
☒	☐	The statistical test(s) used AND whether they are one- or two-sided<br>*Only common tests should be described solely by name; describe more complex techniques in the Methods section.*
☒	☐	A description of all covariates tested
☒	☐	A description of any assumptions or corrections, such as tests of normality and adjustment for multiple comparisons
☒	☐	A full description of the statistical parameters including central tendency (e.g. means) or other basic estimates (e.g. regression coefficient) AND variation (e.g. standard deviation) or associated estimates of uncertainty (e.g. confidence intervals)
☒	☐	For null hypothesis testing, the test statistic (e.g. *F*, *t*, *r*) with confidence intervals, effect sizes, degrees of freedom and *P* value noted<br>*Give P values as exact values whenever suitable.*
☒	☐	For Bayesian analysis, information on the choice of priors and Markov chain Monte Carlo settings
☒	☐	For hierarchical and complex designs, identification of the appropriate level for tests and full reporting of outcomes
☒	☐	Estimates of effect sizes (e.g. Cohen's *d*, Pearson's *r*), indicating how they were calculated

Our web collection on statistics for biologists contains articles on many of the points above.

Software and code

Policy information about availability of computer code

Data collection	X-ray data was collected using in-house software at the Australian Synchrotron MX beamlines. Mass spectrometry - QSTAR (LC-MS): acquisition software Analyst QS v1.1, TripleTOF (LC-MS/MS): acquisition software Analyst TF v1.8. NMR -Software for acquisition: Topspin, v2.1 (Bruker).
Data analysis	X-ray data was processed using XDS and aimless software packages. Mass spectrometry data processing software for both LC-MS and LC-MS/MS: PeakView v2.2. NMR - Software for processing: Topspin, v4.1.4 (Bruker).

For manuscripts utilizing custom algorithms or software that are central to the research but not yet described in published literature, software must be made available to editors and reviewers. We strongly encourage code deposition in a community repository (e.g. GitHub). See the Nature Portfolio guidelines for submitting code & software for further information.

Data

Policy information about availability of data

All manuscripts must include a data availability statement. This statement should provide the following information, where applicable:

- Accession codes, unique identifiers, or web links for publicly available datasets
- A description of any restrictions on data availability
- For clinical datasets or third party data, please ensure that the statement adheres to our policy

Crystal structure were deposited with the Protein Data Bank (PDB; https://www.wwpdb.org/) with accession codes 7ULD, 7ULE, 7ULF, and 8G8P. All other data supporting the findings of this study are available within the paper and its Supplementary Information.

Research involving human participants, their data, or biological material

Policy information about studies with human participants or human data. See also policy information about sex, gender (identity/presentation), and sexual orientation and race, ethnicity and racism.

Reporting on sex and gender	n/a
Reporting on race, ethnicity, or other socially relevant groupings	n/a
Population characteristics	n/a
Recruitment	n/a
Ethics oversight	n/a

Note that full information on the approval of the study protocol must also be provided in the manuscript.

Field-specific reporting

Please select the one below that is the best fit for your research. If you are not sure, read the appropriate sections before making your selection.

☒ Life sciences ☐ Behavioural & social sciences ☐ Ecological, evolutionary & environmental sciences

For a reference copy of the document with all sections, see nature.com/documents/nr-reporting-summary-flat.pdf

Life sciences study design

All studies must disclose on these points even when the disclosure is negative.

Sample size	For all experiments the sample size is n=1. In the X-ray crystallography, data were collected from single unique crystals. In NMR experiments, data were collected from a single solution sample of each reaction over a time line. In mass spectrometry experiments, data were also collected from single solution samples over various time points. In each experiment, statistical averaging of measurements is carried out as appropriate to the methodology.
Data exclusions	In X-ray analysis some data is excluded by the processing software packages XDS and aimless. This is by default using default software settings without user intervention and is standard in the field.
Replication	In optimizing mass spectrometry and NMR, all experiments were replicated several times. In X-ray crystallography, as is the norm in the field, crystal structures were determined once.
Randomization	None of our experiments require randomization.
Blinding	None of our experiments require blinding.

Reporting for specific materials, systems and methods

We require information from authors about some types of materials, experimental systems and methods used in many studies. Here, indicate whether each material, system or method listed is relevant to your study. If you are not sure if a list item applies to your research, read the appropriate section before selecting a response.

Materials & experimental systems

n/a	Involved in the study
☒	☐ Antibodies
☐	☒ Eukaryotic cell lines
☒	☐ Palaeontology and archaeology
☒	☐ Animals and other organisms
☒	☐ Clinical data
☒	☐ Dual use research of concern
☒	☐ Plants

Methods

n/a	Involved in the study
☒	☐ ChIP-seq
☒	☐ Flow cytometry
☒	☐ MRI-based neuroimaging

Eukaryotic cell lines

Policy information about cell lines and Sex and Gender in Research

Cell line source(s)	Sf9 insect cells
Authentication	The Sf9 expression was used as a service provided by the Protein Expression Facility, The University of Queensland, Australia.
Mycoplasma contamination	The Sf9 expression was used as a service provided by the Protein Expression Facility, The University of Queensland, Australia.
Commonly misidentified lines (See ICLAC register)	*Name any commonly misidentified cell lines used in the study and provide a rationale for their use.*
